# Common Criteria Related Security Design Patterns—Validation on the Intelligent Sensor Example Designed for Mine Environment

**DOI:** 10.3390/s100504456

**Published:** 2010-04-30

**Authors:** Andrzej Bialas

**Affiliations:** Institute of Innovative Technologies EMAG, 40-189 Katowice, Leopolda 31, Poland; E-Mail: a.bialas@emag.pl; Tel.: +48-32-2007-700; Fax: +48-32-2007-701

**Keywords:** Common Criteria, IT security development, intelligent sensor, design pattern, methane detector

## Abstract

The paper discusses the security issues of intelligent sensors that are able to measure and process data and communicate with other information technology (IT) devices or systems. Such sensors are often used in high risk applications. To improve their robustness, the sensor systems should be developed in a restricted way to provide them with assurance. One of assurance creation methodologies is Common Criteria (ISO/IEC 15408), used for IT products and systems. The contribution of the paper is a Common Criteria compliant and pattern-based method for the intelligent sensors security development. The paper concisely presents this method and its evaluation for the sensor detecting methane in a mine, focusing on the security problem of the intelligent sensor definition and solution. The aim of the validation is to evaluate and improve the introduced method.

## 1. Introduction

The paper concerns the intelligent sensor security development methodology compliant with ISO/IEC 15408 Common Criteria (CC) [1]. This standard provides assurance for the designed information technology (IT) product or system. Assurance means that the product or system meets its security objectives. In other words, it means that the implemented measures will be able to counter threats when they occur. The sources of assurance are the rigor applied during development and manufacturing processes, independent third-party evaluations as well as operation and maintenance according to the obtained certificate. These specially developed and evaluated IT products (hardware, software, firmware) and systems can be applied in a higher risk environment. The CC methodology has been broadly used for years. More than a thousand certification processes were completed and the certificates deal with varied products or systems [2].

Generally, sensors are devices that measure a physical quantity and convert it into a signal, which can be read by an observer or instrument. A number of such devices contain actuators. Intelligent sensors are able to process measured values and may be organized in sensor systems. They use network technology for integration, including the wireless one. With respect to the CC methodology, intelligent sensors can be considered as IT products. They have hardware and software components, co-operate with other IT systems and, first and foremost, the IT inherent risks should be considered for them.

The number of intelligent sensors implementations is growing, though the number of finalized Common Criteria certification processes of these IT products is relatively low. The intelligent sensor systems application can be discussed as one of the emerging application domains of the Common Criteria standard.

The motivation of this paper is to provide the developers with knowledge and a set of validated design patterns related to the application of the Common Criteria methodology to the intelligent sensors development. This can help them in a broader use of this methodology in dependable sensor solutions, including sensors designed for the mine environment.

This paper is a continuation of [3], which includes:

- the primer of the Common Criteria standard designed for sensor systems developers;
- the review of the author’s earlier researches leading to the elaboration of a CC-based, IT security development method focused on the intelligent sensors and sensor systems;
- the discussion of architectures, applications and security issues of intelligent sensors and sensor systems; reviewed security works, focused on attack analysis and an advanced security mechanism, do not provide a method of implementation of these mechanisms with the use of assurance methods, for example Common Criteria; this discussion allows to propose two models: a generalized intelligent sensor model and, related to it, CC-compliant intelligent sensor security model;
- the Common Criteria related security design patterns, elaborated on the basis of the exhaustive analysis of security features and behaviors of different kinds of sensors working within varied operational environments; these patterns can be used to fulfil the security model with contents specific to a given sensor;
- the validation of this set of patterns on the intelligent medical sensor example elaborated on the basis of the [4] paper.

The elaborated Common Criteria related security design patterns (shortly: “CC-related patterns”) [3] should be validated on varied sensors projects to optimize this set and its particular items. The set should contain necessary and sufficient items to express common security features and behaviors of intelligent sensors in their most representative application domains. Particular items should have a general, universal meaning, allowing their use in a broad range of applications. Thanks to introduced operations on patterns (called “enhanced generics”, explained later), like: refinement, parameterization or iteration, different details can be added to their contents, allowing them to address very specific security issues. To elaborate an adequate, optimized set of patterns, different validations on varied projects are planned. More optimized patterns improve the usability and quality of designs.

The contribution of the paper, related to the [3] contribution, is to provide a CC-compliant, pattern-based IT security development method focused on intelligent sensors.

The objectives of the paper are:

- to provide a complete, concise description of the pattern-based method,
- method validation on the example of the intelligent sensor detecting methane in a mine,
- method improvement based on the validation results.

The indirect, far-reaching objective is to provide such improved, ordered patterns and related knowledge to a broader group of developers of intelligent sensors or sensor systems designed for high risk environments.

The elaboration of CC-related patterns requires validations on varied projects. Currently, two validations are being finalized: the motion sensor of digital tachographs [5] and the medical sensor remotely monitoring patients [3]. This paper presents another project example—the intelligent sensor detecting methane in the coal mine environment.

The paper includes the following key sections. Section 2 provides a general introduction to the Common Criteria IT security development process, presenting the CC methodology background and the author’s researches on the automation of this process. Section 3 reviews the author’s provided CC-compliant method of intelligent sensors security development. The section presents a general sensor model, its security model and a set of predefined Common Criteria related security design patterns to fulfil this security model with contents specific to the elaborated type of the sensor. The central part of the paper is Section 4 which presents a method validation on the intelligent sensor detecting methane. It is shown how the predefined patterns can be applied to work out the intelligent sensor security specification. Section 5 concludes the validation example and summarizes the methodology as a whole.

## 2. Common Criteria Compliant IT Security Development

The security patterns evaluated here are closely related to the assurance methodology specified in the Common Criteria standard (ISO/IEC 15408). This methodology can be applied to provide dependable IT products or systems for applications, where the assessed, IT inherent risk is significant.

Assurance, measurable in the range EAL1 to EAL7, is identified as the confidence that an IT product or system meets security objectives specified for it. In other words, it means that the built-in security functions related to these objectives and representing measures will work effectively whenever a threat occurs. The source of assurance is rigorous development and independent evaluation, so these IT products or systems are called TOE (target of evaluation). The development encompasses two processes.

The first process, called the IT security development, summarized in the security target (ST) specification includes seven stages [1]/Part 1, but the following five are most relevant:

- Work-out of the “ST introduction”, containing different identifiers, informal TOE overview and TOE description;
- Security problem definition (SPD); SPD specified threats, OSPs (organizational security policies) and assumptions for the TOE and its operational environment;
- Solution of this problem by specifying security objectives (SO)—for the TOE and its operational environment;
- Work-out of the security functional requirements (SFRs) specification on the security objectives basis, and the set of security assurance requirements (SARs) which are derived mainly from the declared EAL;
- Creating the TOE summary specification, including the TOE security functions derived from the SFRs; they are implemented in the TOE on the claimed EAL during the TOE development process.

The paper concerns the first three issues and discusses how to specify elaborated TOE on general level, how to specify the security problem and its solution using the predefined CC-related patterns. Please note that the security target for the TOE [1]/Part 1 can be considered as a security model of the developed IT product or system. Here discussed security patterns concern SPD and SO. For security functions some patterns are predefined too, but only briefly mentioned in the paper. Security functional components [1]/Part 2 and security assurance components [1]/Part 3 play a role of patterns for the security requirements (SFRs, SARs).

The second above mentioned development process, called the TOE development process, is responsible for the implementation and documentation of the elaborated security functions (specified in the ST) on the claimed EAL. Evidences deal with:

- TOE architecture, its functional specification, design, security policy, implementation,
- life cycle definition, configuration management, product delivery, development process security, used tools and their options,
- tests specification, test depth and coverage,
- product manuals and procedures,
- vulnerability assessment of the TOE and its development site.

The TOE, ST, and related documentation (evidences) are provided for the independent evaluation process, finalized by getting a CC certificate for the IT product or system with respect the claimed EAL [2]. More information about the CC methodology can be found in [2], [3]/Section 3 and in the books [6,7]. Table 1 contains the explanation of the terms and acronyms often used in the paper.

The paper is related to the author’s more extensive works aiming at the improvement and automation of the CC methodology by introducing a semiformal description, supporting software tools and by using the knowledge engineering methodology, discussed also in [3]. First, a Common Criteria compliant, UML/OCL-based IT security development framework (ITSDF) [7–9] was elaborated. The framework encompasses:

- models of data structures and processes of IT security development stages,
- models of the specification means used for these IT security development stages, including CC components and the author’s defined semiformal enhanced generics.

The introduced enhanced generics play a role of here discussed CC-related design patterns. They are derived from “generics” commonly used by developers, but are more formalized and can be implemented in software tools. An enhanced generic is defined as a mnemonic name expressing common features, behaviors or actions related to the IT security development process, *i.e.*, subjects and other active entities, assets and other passive entities, threats, assumptions, security policies, security objectives, and functions. They are “enhanced” since they are semiformal and have features comparable to CC components, allowing such operations as: parameterization, derivation, iteration, and refinement.

There are about 350 predefined enhanced generics and they can be applied for commonly used IT products or systems, but the intelligent sensors or their systems, as specific IT products, are not represented there. The CC-related design patterns for this emerging domain of the Common Criteria standard application has been elaborated [3,5], and this paper discusses their validation on the intelligent methane sensor for mines.

## 3. Use of the Common Criteria Related Security Design Patterns for the Intelligent Sensors Development—Concise Method Presentation

The term “Common Criteria related security design patterns”, introduced in [3], needs certain explanation to distinguish them from commonly used design patterns or security design patterns. The CC-related patterns are security design patterns of specific character. They are focused on Common Criteria compliant IT security development, not on IT secure solutions.

Generally, design patterns are reusable, proven solutions to problems with respect to a specific context. They are often used by engineers in many technology domains, including IT- and IT security domains. The patterns provide knowledge and are related to the process that allows achieving the expected solution. Numerous patterns concern software architecture. The patterns are specified in a formalized way, e.g., using UML (Unified Modeling Language) optionally supported by OCL (Object Constraints Language), using different kinds of codes, ontologies, formalized descriptors, *etc*.

The general overview of the software security patterns is presented in [10], which is focused rather on the survey of approaches to security patterns than on the presentation of patterns for given applications. Security patterns are categorized with respect to the software life cycle phases:

- the requirements phase—patterns, e.g., for assets valuation, vulnerabilities and threats assessment, for supporting identification of the security requirements being a part of the system requirements,
- the design phase—patterns related to decisions on conceptual architecture, allowing to select solution variants, concerning: identification, access control, data transfer, audit, non-repudiation, *etc.*
- the implementation phase—to develop secure software without implementation flaws; secure coding techniques and specialized knowledge are provided, which can be considered as security patterns too.

Categorized software patterns, including security patterns, may be placed into repositories available for software developers. In the paper [10] the patterns effectiveness, usability, quality, sufficiency for designs are discussed as well.

The book [11] summarizes researches on the UML extension called UMLsec, providing a unified approach to security features description during the secure systems development. UMLsec can be used to evaluate UML specifications against vulnerabilities. The established formal rules of security engineering can be encapsulated, and hence made available to a wider group of developers. UMLsec allows definition of the UML patterns that encapsulate the design knowledge in the form of recurring design problems, and consist of the “pattern name”, “problem description”, “problem solution” and “consequences”. These patterns deal with many security engineering solutions, like: secure channel, TLS Internet protocol, electronic purse, secure Java programs, bank applications, biometric authentication systems, electronic signature, *etc.* These works focus on modeling IT security features and behavior within the system, and not on the IT security development process compliant with the Common Criteria standard. Relationships between CC-related patterns presented by the author of this paper and the UMLsec patterns defined in [11] are discussed in the monograph [7]. Please note that patterns are a commonly used term in the UML world.

The book [12] presents security design patterns related software solutions, which can be mostly considered as architectural patterns. These solutions, mainly related to enterprise management applications, may concern e.g., enterprise security and risk management, identification and authentication, access control models and systems or operating system access control, accounting facilities, firewall architecture, secure Internet applications, IP telephony and cryptographic key management. Regarding the Common Criteria methodology, this kind of security design patterns is related to the security functions implemented within an IT product or system.

Here discussed security patterns and the method of their implementation are closely related to the risk issue, *i.e.*, assets, legal subjects, attackers, threats, security policies, assumptions and security objectives, of the Common Criteria compliant IT security development process.

To sum up, the presented Common Criteria related security design patterns can be considered as a specialized kind of design patterns because:

- they concern the design process (called here IT security development),
- they can be used in many projects (*i.e.*, they are reusable) but in a certain context (*i.e.*, in the CC-methodology context).

Generally, the patterns represented by the enhanced generics (Section 2) can be applied for different kinds of IT products or systems but the paper is focused only on the patterns subset related to sensors and sensor networks.

### 3.1. General Intelligent Sensor Model

The CC methodology needs a subject for discussion, *i.e.*, an IT product or system being analyzed and secured during the IT security development process. This “subject” is expressed by the generalized IT product or system model, here the “intelligent sensor model”, characterizing a broader group of such sensors.

The initial stage of the IT security development process is the “ST introduction”, containing:

- the “TOE overview”, presenting the “TOE Usage and major security features”, “TOE type” and “Required hardware-, software- or firmware elements in the TOE environment”,
- the “TOE description” presenting the “Physical scope of the TOE” and “Logical scope of the TOE”.

**Figure 1.**
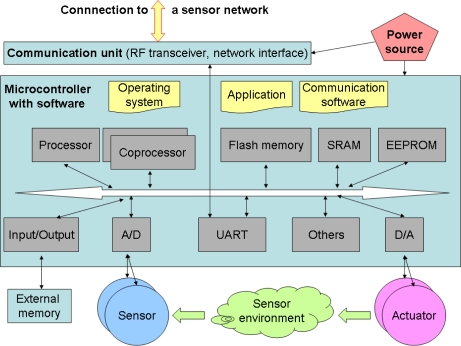
Generalized model of the intelligent sensor used for security analyses during the IT security development process [3].

The discussed intelligent sensors security development method should present how to specify these issues for a broader class of these devices. Reviewing scientific and technical papers, standards, security targets of certified products, technical documentations of leading providers and application notes, the common features of the intelligent sensors and sensor systems are identified and specified in the form of a general intelligent sensor model co-operating with other hardware or software entities of its operational environment. This way, a general, block scheme level model is developed, which creates the context for further security analyses leading to the security functions work-out. The considered general model of the sensor includes the following elements (Figure 1 [3]):

- “microcontroller, equipped with a specialized operating system, communication and application software, different devices, such as memories, including non-volatile memories, interfaces, timers, counters, sometimes other coprocessors (e.g., crypto-processors, network processors and other specialized processors), interfaces to external memories, and other devices (I^2^C, SPI),
- low-power transducers (varied sensing/controlling devices),
- communication facilities, including low-power wireless facilities or traditional/industrial network interfaces (wired, optical facilities),
- power source (battery, solar, external sources)”.

Please note that the intelligent sensor is able to measure physical values, process them and communicate with other co-operating nodes and end-user applications. Optionally, intelligent sensors are provided with actuators. Restricted resources related to processing, transmission and energy should be considered during security analyses, because restrictions influence negatively the sensor capability.

The general model can be used as a reference model for a broader class of considered intelligent sensors. The important issue is to identify what parts belong to the TOE (it depends on the project) and what parts co-operate with the TOE, being elements of its operational environment. This abstract level should be enough to present boundaries of the TOE, which elements are parts of the TOE, and which are parts of its operational environment. Moreover, this model can be used as a background to present the basic TOE user’s functionality, protected assets, their owners, intruders, threats, security policies, connectivity aspects, and security objectives.

### 3.2. Notation of the CC-Related Security Design Patterns

The author’s defined enhanced generics [7–9] play a role of CC-related security design patterns. This notation concerns all kinds of IT products or systems, not only intelligent sensors. To make the reading of this paper easier, let us refer to the enhanced generics notation from [3], which is very similar to the notation of the security components (SFR/SAR) specified in the [1] standard.

In comparison with the patterns specification in [3], the following changes of the enhanced generics semantics were provided:

- the “TOE IT environment” is understood as the “TOE operational environment”, including IT-, physical-, personnel- and organizational aspects of this environment;
- the former “TOE physical environment” concerns the development-, manufacturing- and maintenance processes performed within the certain “site”, which may be distributed or not.

To better express the current situation some prefixes were redefined (see subsection 3.2.1 below):

- “DIT” to “DEO”, “TIT” to “TEO”; “EO” means: “Environment–Operational”;
- “DAP” to “DES”, “TPH” to “TES”; “ES” means: “Environment–Site”.

The TOE operational environment encompasses technical and procedural facilities in the TOE neighborhood, enabling the TOE operation, including measures assisting the TOE in correctly providing its security functionality [1]/Part1.

The “site” can be considered as the certain TOE environment, placed “far from the TOE” but logically related to it, because the TOE was developed and manufactured there and the TOE can be maintained there. The site can be distributed or not and it encompasses technical and procedural facilities enabling the TOE development-, manufacturing- and maintenance processes. It should include measures protecting these processes. Removing vulnerabilities related to the site processes, influences positively the TOE in the operational environment.

This revision improves generally the compatibility with the latest Common Criteria 3.1 version, according to which the ST security analyses are focused on the TOE and its operational environment only. Different security issues related to the development-, manufacturing- and maintenance processes should not be explicitly specified, as in the older security targets, e.g., [13,14].

In the presented method, the security issues dealing with the site processes can be specified optionally, being useful in the security models considering all phases of the life cycle model, not only the operational phase. The full life cycle model is convenient for the IT products like RFID/smart cards and sensors, and is compliant with the latest researches on the site certification [15]. It is useful to consider the security issues related to the site e.g., assets, threats, assumptions, *etc.*, or sometimes it is even required for the above mentioned IT products. Unsolved security problems in the site may cause security problems for the TOE. The optionally identified security problems concerning the development-, manufacturing- and maintenance processes are considered in the TOE development process with respect to the assurance requirements included in the assurance families: ALC_DVS (Life-cycle–Development security) and AVA_VAN (Vulnerability assessment–Vulnerability analysis) [1]/Part3.

Due to the performed changes, a complete, revised specification of the enhanced generics is here provided (denoted in author’s project as the “Enhanced generics rev.3.0”). An enhanced generic consists of four textual fields separated by dots, and the fourth field *Refinement* is optional:

Family.Mnemonic.Description.Refinement

#### 3.2.1. Field Expressing Generics Family

There are several items in the *Family* field. Each security model issue, like: assets, subjects, threats, *etc.*, has its own possible values. Families are represented by particular mnemonic prefixes.

The CC-defined functional paradigm [1]/Part 2 says that an asset represents a passive entity within the considered system. The *Family* field for assets (data, services, software, IT infrastructure, documents, *etc*.) begins with “D”, and the following values are allowed (the symbol “|” means “or”): *Family* ::= DTO | DEO | DES. Family semantics for passive entities is defined in Table 2.

Subjects, representing active entities related to the TOE, the TOE operational environment and optionally the site, are preceded by prefixes: *Family* ::= SAU | SNA | SNH. Family semantics for active entities is shown in Table 3.

The following prefixes are defined for threats: *Family* ::= TDA | TEO | TES. Family semantics for threats are presented in Table 4.

Assumptions, addressed to the TOE operational environment and optionally to the site, have prefixes, expressing aspects: *Family* ::= ACN | APR | APH. Family semantics related assumptions are defined in Table 5.

Similar prefixes are defined for security objectives:

*Family* ::= OACC |OIDA |OADT |OINT |OAVB |OPRV |ODEX |OCON |OEIT |OEPH |OSMN and for organizational security policies (OSPs):

*Family* ::= PACC |PIDA |PADT |PINT |PAVB |PPRV |PDEX |PCON |PEIT |PEPH |PSMN. Family semantics for both issues are defined in Table 6.

As it was stated above, the security functional requirements (SFRs) are specified on the basis of the TOE security objectives. The security functional components defined in [1]/Part 2 can be considered as CC-related design patterns to specify the SFRs. These components are organized by functional classes, and classes by their families, grouping similar issues expressed by functional components.

It should be noted that enhanced generics may express security functions as well. The paper does not discuss their elaboration, but they will be included below in Table 7 to present a complete set of predefined enhanced generics used as CC-related design patterns. Prefixes of the security functions begin with the letter “S”, and further include a CC functional class name which the given function deals with, e.g., for the “FAU” class (audit), the security functions have the “SFAU” prefix. For the security functions the following prefixes are defined:

*Family* ::= SFAU |SFCO |SFCS |SFDP |SFIA |SFMT |SFPR |SFPT |SFRU |SFTA |SFTP. Family semantics for security functions are described in Table 7.

#### 3.2.2. Field Expressing the Mnemonics of a Generic

The second enhanced generics field, developers-defined *Mnemonic*, expresses very briefly, in a few letters, the generic meaning (see examples below in Tables 8–16).

#### 3.2.3. Field Presenting Description (Meaning) of the Generics

The *Description* field presents concisely, in one or few sentences, the generic meaning, *i.e.*, the security features, behavior or actions. This field may have parameters in square brackets representing any asset [*Dparam*] or any subject [*Sparam*]. Parameters may be left empty (meaning: “any possible”) or substituted (symbol: “<=”) by an appropriate asset or subject generic. Parameterization enables to perform the iteration of enhanced generics. In this case the given generics can be placed many times into the specifications with different parameters substituted, and presenting different aspects of the same issue. For example, the threat item with intruders of different potential attacking the same asset, or the threat with one intruder attacking different assets in a different way. Particular instances of the iterated enhanced generic are numbered and their consecutive numbers are in brackets.

#### 3.2.4. Field Refinement Supplementing Description of Generics

Please note that the content of the *Description* field is predefined for enhanced generics placed in a tool library or knowledge base. If the given generics are used in the project, some extra information can be added on the project level by means of the last, optional field, called *Refinement*. As it was the case with CC components, this word is placed below the generic description as an underlined word *Refinement*, which will be shown in the examples below (subsections: 4.2−4.4).

### 3.3. The CC-Related Security Design Patterns Defined for the Intelligent Sensor Application Domain

The above presented enhanced generics notation is used for generics of different application domains and these generics can be considered here as CC-related design patterns for a given domain.

The CC-related design patterns for the intelligent sensor and sensors system domain were defined in the paper [3] and validated on the medical sensor example. Different security issues were analyzed and generalized into the form of particular generics. The following inputs discussed there in details were used:

- broad range of publications discussing the WSN (Wireless Sensor Networks) specific problems and solutions, like: known methods of attacks, resource limitations, architectures and software constraints, application specific issues for MANET (Mobile Ad-hoc Network) and VANET (Vehicular Ad-hoc Networks),
- publications presenting emerging Common Criteria applications dealing with airplane health monitoring systems, safety-critical assets distribution systems, motion sensors of digital tachographs, SCADA (Supervisory Control And Data Acquisition) related products and specialized firewalls used in control and automation systems, co-operating with sensor networks,
- publications concerning intelligent sensors working autonomously or integrated using the traditional network technology (a cooper wire, a fibre optic),
- security targets of certified IT product or systems, used life cycle models for different IT products,
- papers and security targets devoted to the smartcard and RFID (Radio Frequency Identification) technologies; this is a mature domain of the Common Criteria application but security problems are very similar to the intelligent sensors domain, considered as an emerging one.

On this basis, a set of Common Criteria related security design patterns were elaborated. They are grouped according to categories existing in the IT security development process, *i.e.*, assets, subjects, threats, *etc.*, presented in separate subsections below.

### 3.4. Patterns Expressing Assets and Other Passive Entities

According to the CC functional paradigm [1]/Part 2, assets are passive entities which should be protected by the TOE. Assets can be included in the TOE, e.g., secret key within the sensor microcontroller internal register, or can be placed outside of the TOE, e.g., the data sampled by sensors and stored in the data base placed in the WSN base station. Assets are managed by authorized active entities (owners, users, administrators) or attacked by unauthorized active entities (intruders)—both entities represented in the method by subjects—discussed in the next subsection. Assets may have values assigned to assess risk (optional) when threats occur. Assets are used as parameters values in other generics, except subjects, especially in threats and OSPs. Assets may be sampled, processed, stored or transmitted data, services provided, IT elements or physical elements.

Table 8 presents enhanced generics expressing different kinds of intelligent sensor assets:

- basic assets, *i.e.*, data sampled, processed, stored and transmitted, and the provided services,
- assets whose availability allows the right operation of sensors (please note the restricted resources–energy, processing- and transmission capability),
- security-related data,
- assets placed within the TOE operational environment, encompassing all co-operating and mutually related IT entities,
- assets related to the TOE development-, manufacturing- and maintenance processes, encompassed by the life cycle model, placed “somewhere far” from the TOE, *i.e.*, in the site (considered optionally).

### 3.5. Patterns Expressing Subjects and Other Active Entities

According to the CC functional paradigm [1]/Part 2, the active entities are distinguished. They express varied “actors” operating around the TOE. Most of them are subjects, having their own logical representations within the TOE, *i.e.*, legal users/asset owners (human users, or non-human users, e.g., processes). Apart from legal users, the intruders trying to breach or destroy the protected assets are identified. Legal users may also breach assets, intentionally or not. Additionally, special kinds of intruders representing non-human entities, events or actions causing incidents are considered.

The most important subject related to the intelligent sensor system represents a sensor user, who/which can take different forms depending on the user’s nature. The personnel, considered as the sensor user, communicates with the sensor indirectly through the base station and the network where the sensor works. Sometimes the sensor user can be considered as a process communicating directly with the sensor to get some data or to perform some operations. Moreover, the administrator of the network assets and applications is distinguished and – optionally – other roles in different phases of the life cycle in the site.

Table 9 presents enhanced generics expressing different kinds of active entities related to the intelligent sensor, *i.e.*:

- legal users of the sensor or sensor system,
- legal actors participating in the life cycle processes performed within the site,
- intruders,
- intruders relevant to the site processes.

### 3.6. Patterns for Threats Representation

The enhanced generic representing a threat consists of three elements:

- a threat agent, which adversely acts on an asset, e.g., hacker, user, developer, process, force majeure, failure; in the presented methodology it can be expressed by the Sparam parameter substituted by a subject-type generic from Table 9;
- attacked asset (has value for its owner, so it needs protection), e.g., data, provided IT service, ability to use the IT equipment; in the presented methodology it can be expressed by the Dparam parameter substituted by an asset-type generic from Table 8;
- adverse action performed on an asset by a threat agent who exploits existing vulnerabilities (a threat scenario).

The given threat can be placed into the specification with different parameters substituted or refined in a different way. It enables iterations providing more compact and precise specifications.

The threats specification is the key part of the security problem definition. Threats should be countered by the TOE, *i.e.*, the intelligent sensor, or by its operational environment, both partitioned by the TOE boundaries described in the TOE description (please note the logical/physical scopes). The third group of threats, related to the development-, manufacturing- and maintenance environments, influences indirectly the TOE.

The first, main group of threats (Table 10) encompasses direct attacks against the TOE:

- related to the data sampled/measured by the intelligent sensor,
- related to the data stored, processed and transferred by the intelligent sensor,
- exploiting vulnerabilities concerning sensors restricted resources,
- aimed at the sensor identity,
- trying to breach the security-related data,
- aiming at the sensor integrity,
- related to different cases of unforeseen natural catastrophes, emergencies and failures.

The intelligent sensor operational environment encompasses IT elements, like neighbor sensors, network nodes, *etc.* with their hardware and software co-operating with the TOE. Attacks against the TOE operational environment, shown in Table 11, can impact the TOE indirectly.

With respect to the life cycle model, the above two groups of threats concern the TOE operation. Other phases of the life cycle model are expressed by the third group, generally concerning the site and its development-, manufacturing- and maintaining processes (Table 12). They indirectly impact the TOE.

### 3.7. Patterns Related to the Organizational Security Policies

According to the Common Criteria methodology [1]/Part 1, the Organizational Security Policies (OSPs) encompass security rules, procedures or guidelines, existing or imposed to implement in the TOE operational environment by the organization which deploys the TOE. Generally, the OSPs are enforced by the TOE or the TOE operational environment. Optionally the OSPs enforced in the site can be specified.

Please note that OSPs are varied. Sometimes they can express simple information flow rules or access to asset rules, e.g., DAC—Discretionally Access Control, MAC—Mandatory Access Control, sometimes they imply standard- or regulations compliance or the use of certain procedures.

Table 13 shows the Organizational Security Policies related to the TOE, its operational environment and site processes.

The security developers’ experiences show that the threats-based security problem definition gives more compact and concise specifications influencing the developed IT product or system quality. “The protection against the identified threats” is preferable by developers. The OSPs are used to specify issues hard to express by threats, as those listed in Table 13.

### 3.8. Patterns Representing Assumptions

Assumptions are specified for the TOE operational environment in order to show what conditions should be satisfied to enable the TOE security functionality. If the TOE placed in a certain operational environment does not meet these assumptions, the TOE may not be able to provide all of its security functionalities any more [1]/Part 1. Assumptions express personnel-, physical- and connectivity aspects of the operational environment, though for the intelligent sensors only these expressing the personnel (including organizational ones) aspects (APR) have been defined up until now (Table 14). Assumptions can never be made on the TOE behavior. Optionally, some assumptions are predefined for site processes.

Assumptions are transformed to the appropriate security objectives for the operational environment that upheld these assumptions. Generally, the efficacy of the countering threats and the efficacy of the enforcing OSPs depend on satisfying assumptions addressed to the TOE operational environment. Assumptions for site processes represent certain developer demands concerning site processes and are transformed into security objectives related to the site.

### 3.9. Patterns for Security Objectives

The security objective represents a concise and abstract statement of the intended solution of the given elementary problem defined in the security problem definition of the security target. Security objectives, formulated in a natural language, should be understandable to knowledgeable potential consumers of the TOE [1]/Part 1/. The specified intended solution partially deals with:

- the TOE, specified as the TOE security objectives,
- the TOE operational environment, expressed by the security objectives for the operational environment,
- the TOE site (optionally), specified with the use of security objectives concerning mainly the site processes.

Table 15 contains appropriate groups of the security objectives enhanced generics concerning:

- mainly the TOE,
- mainly the TOE operational environment,
- the development-, manufacturing-, and maintenance processes performed in the site.

The word “mainly” says that using the given item depends on the situation, the project character.

TOE security objectives express how the TOE counters threats and/or enforces OSPs. Later, these objectives are transformed to the security functional requirements and are implemented in the TOE security functions. Security objectives for the operational environment express how the threats are countered, OSPs are enforced and assumptions are upheld in this environment.

### 3.10. Patterns for Security Requirements

The security requirements encompass two groups:

- the security functional requirements (SFRs), derived from the TOE security objectives, *i.e.*, these objectives are translated into the standardized, semiformal CC language (SFR components) components [1]/Part 2;
- the security assurance requirements (SARs), presenting how assurance is to be gained that the TOE meets SFRs; SARs are usually implied by claimed EAL and expressed by the semiformal SAR components [1]/Part 3.

There is no difference between approaches to the intelligent sensors security requirements and other IT product or systems.

The SFR- and SAR components can be considered as the CC-related patterns to specify requirements for intelligent sensors. There are no specific issues related to the considered here Common Criteria domain of application.

### 3.11. Patterns for TOE Security Functions

The TOE security functions (TSFs) specified in the TSS, *i.e.*, the TOE summary specification (the final part of the ST) expresses how the TOE satisfies the SFRs. The TSS provides the general technical mechanisms that the TOE uses for this purpose [1]/Part 1.

This paper, as well as [3], does not discuss the full security target elaboration, showing only two initial and application specific stages, which often make difficulties for developers. The enhanced generics of the TSFs, presented in Table 16 can be considered as examples of definitions, rather than patterns. They concern the intelligent motion sensor example of digital tachograph compliant with the directive [14], still being able to express some key issues of other kinds of sensors. This part of the presented method needs further researches.

## 4. Validation of the CC-Related Design Patterns and Related Security Development Method

The subject of validation concerns the mining applications of intelligent sensors. Mining sensors are specific in comparison with industry sensors, like [17,18]. Rock and methane outbursts, as well as rock bumps, are the most dangerous and still poorly predictable phenomena in hard coal mining. Early detection of such hazards is extremely important for the mine crew safety and the mine operation. The robust, dependable IT-based solutions should be applied in this domain. The intelligent sensors which are key-importance elements of hazards early detection systems, and these systems as such, are IT based and designed for high risk applications, so the Common Criteria methodology can be applied for them.

There are different kinds of systems developed for mine environment monitoring, equipped with varied sensors, like methane detectors, carbon monoxide analyzers, smoke detectors, oxygen meters, air temperature sensors, anemometers, seismic sensors, coal dust detectors, *etc.* Monitoring systems can work autonomously but usually they are integrated in a broader IT system co-operating with the mine telecommunications system, including loud speaking communication and alarm signaling, and the mine SCADA system which monitors varied safety and production parameters in the mine. Such systems and different types of intelligent sensors have been developed and offered by the author’s organization for years [19].

The paper [20] presents two variants of mine monitoring systems. The first allows the monitoring of varied safety and production parameters of a mine, for early detection of a fire, to support ventilation service, to switch off electric power in the mine monitored area in the case of an emergency or breakdown. The second, more complex, is integrated with an alarm-loud-speaking/signaling system and is connected to the mine SCADA system. In both cases, the further discussed intelligent sensor can be considered as one of the sensors working at the lowest level of the system hierarchy.

The paper [21] discusses an intelligent, integrated sensor detecting methane and rock outbursts, measuring methane concentration, pressure wave in the excavation and the acoustic effects of the outburst simultaneously. The paper analyzes transient states of air parameters considering input data sampled during two serious, real events related to methane and rocks outbursts which have occurred in Polish mines recently. The results of analyses are used to develop the integrated sensor. Early detection of higher concentration of methane requires right dynamic parameters of the applied pellistor-type methane measurement element. The model of the integrated sensor, its technical data and construction tests are discussed in this paper.

The CC-related security development method presented in the paper is validated on the intelligent sensor example designed for early detection and signaling of methane hazards in coal mines. This intelligent sensor, operating in a high risk environment, integrated with a broader monitoring system, represents a new class of IT product, which can be designed with the use of the Common Criteria methodology. Besides, the safety issues, including IT security issues, are important for such devices, because one cannot disregard threats inherent to the applied IT solutions in the sensor and threats related to the IT systems co-operating with the sensor.

The aim of the validation is to determine whether the predefined security design patterns can be applied for the considered design case. Validations should optimize the set of patterns and improve its particular items. During the validation, the predefined design patterns will be used to express the security problem definition and its solution. To build the security specifications, *i.e.*, threats-, OSPs-, assumptions specification, *etc.*, first the best matching enhanced generics from the predefined ones will be identified. To express specific issues, they can be refined. If a given elementary security problem or its elementary solution cannot be expressed this way, a new enhanced generic should be defined and added to the set of patterns. An important issue is to avoid defining redundant patterns.

The example deals with the Methane Early Detection Intelligent Sensor (MEDIS), working within the broader Mine Monitoring System (MMS), controlling different safety and production parameters, e.g., physical parameters, chemical composition of air, as well as the condition and working parameters of ventilation equipment, machines and process-line equipment. MMS is designed for mines with methane and coal-dust explosion hazards. The MMS system co-operates with the mine SCADA and integrates MEDIS devices and other sensors measuring different mine atmosphere parameters. MMS allows detecting and signaling of natural hazards, particularly methane- and fire hazards, high-energy rock bursts, along with other hazards related to production processes. In the case of an event, the energy supplied for the mining equipment in the monitored zone is switched off. MMS can be integrated with the alarm and communication system of the mine, enabling automatic transmission of voiced alarm and evacuation messages which inform the personnel about the existing danger and evacuation ways. MMS should comply with the ATEX directive and mining regulations.

The validation is focused on the selected issues of the elaborated security target (ST) of MEDIS, considered here as the target of evaluation (TOE). The validation is based on the general model and the security model (*i.e.*, ST) of the intelligent sensor presented in [3]. The MEDIS security target is elaborated with the use of here validated, predefined design patterns.

The example discusses how to elaborate key parts of the MEDIS security targets. First the “ST introduction”, defining the TOE, its parts and its operational environment is shown. On this basis the security problem definition, embracing threats, policies and assumptions, will be formulated. The security problem is solved by specifying security objectives, which are later transformed into security requirements, encapsulated in the security functions implemented within the intelligent sensor.

Please note that the example concerns not only the security problem definition but also its solution, and these issues are presented here together, in a slightly different way as in the real security targets. To better explain the presented issues, the security objectives (elementary solutions) are specified immediately when a given elementary security problem (threat, OSP, or assumption) is identified. The security target documents are rather extensive [2]. The full ST for the intelligent sensor cannot be discussed here in detail. The paper discusses within the selected issues how to elaborate it.

### 4.1. Identifiers and Informal IT Product Description Included in the “ST Introduction”

**Figure 2.**
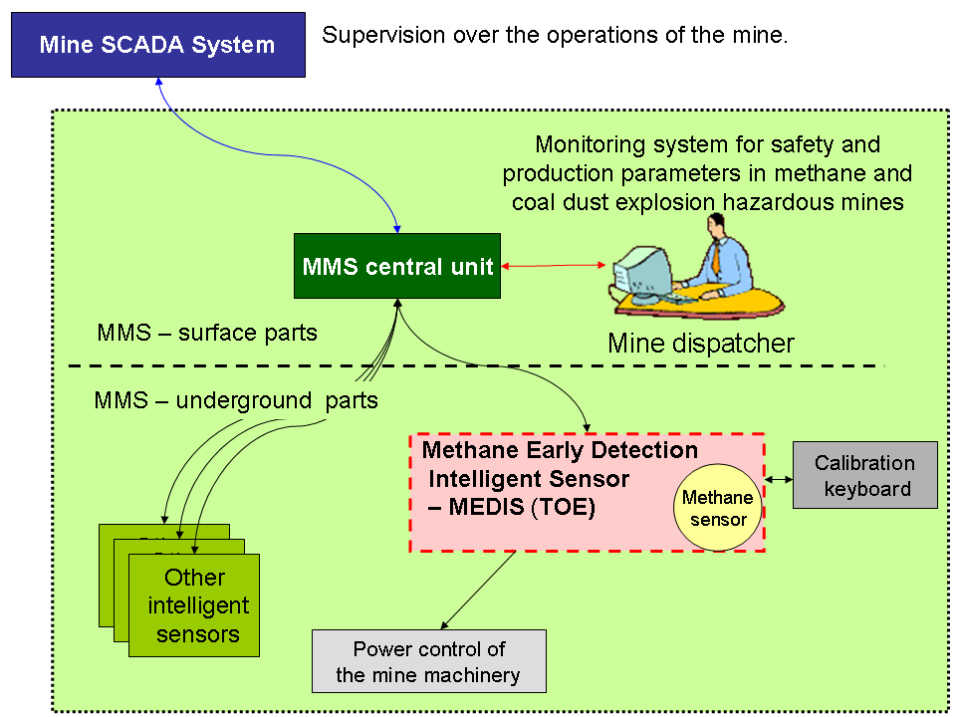
General architecture of the “Mine Monitoring System (MMS)”.

The “ST introduction” contains:

- the TOE identifier, e.g., “*MEDIS—Methane Early Detection Intelligent Sensor, ver. 0.9*”,
- the ST identifier, e.g., “*ST for the MEDIS ver. 0.8*”,
- the “TOE overview” and more detailed “TOE description”,

where the basic TOE features and components are described. For the discussed MEDIS sensor, the TOE description can be expressed as follows:

*“The MMS system (Figure 2) is designed for distance, on-line monitoring of mine atmosphere, including methane. The mining environment, especially ways and long walls are equipped with different kinds of sensors, such as: methane sensors* (i.e., *the MEDIS type—TOE), carbon monoxide analyzers, smoke detectors, oxygen meters, anemometers, air-temperature meters,* etc.

The measured values of the mine environment parameters are transmitted over the wire connections to the MMS central unit, where they are processed and supervised by the mine dispatcher. The central unit co-operates with other mine supervising systems including the SCADA system. The intelligent MEDIS sensors are supplied by the central unit or an equivalent power source, when the MEDIS operates autonomously. In the case of a methane hazard MEDIS is able to cut off the power of the mine machinery in the monitored zone. The mine service personnel provide periodical inspections and calibration with the specialized calibration keyboards.”

**Figure 3.**
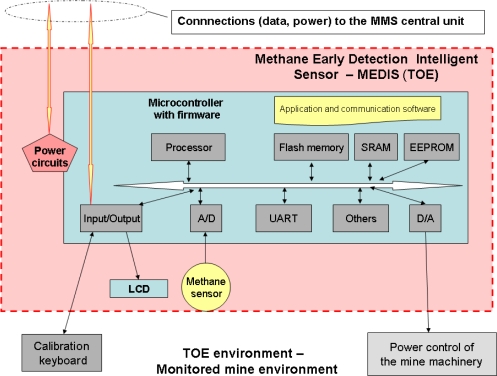
MEDIS—Methane Early Detection Intelligent Sensor as the TOE.

One of important issues is to present what the TOE is and what is in the TOE operational environment, defining the TOE logical/physical components and the TOE boundary, e.g., (continuation of the “TOE description”):

*“The TOE (target of evaluation) (Figure 3) consists of a microcontroller with software, wired external MMS interface, methane sensor with its own microcontroller responsible for measurement, LCD type display, battery and power circuits, digital interface of an optionally connected (while the sensor is serviced) calibration keyboard and an analogue interface to control the power lines of the mining equipment which operates within the monitored environment. MEDIS is able to work autonomously but in this case it is powered by an equivalent power source and is unable to cut off the power of the mine machinery working within the monitored environment. The TOE also includes the application and communication software (firmware) running in the microcontroller. The MEDIS sensor provides the following main users functionalities:*

─ communication with the MMS central unit working on the surface area,
─ monitoring if methane concentration exceeds assumed thresholds,
─ issuing an optical signal in the case of a gas alert or faults,
─ self testing.

The TOE boundary is marked with dashed lines. Physically, the TOE encompasses two special, reinforced cases. The first of them includes electronic circuits on a few PCB modules allowing configuring different versions of the TOE, the second—all external connections. Authorized service personnel periodically calibrate methane sensors and set up the alarm thresholds (when the power should be switched off).”

### 4.2. Security Problem Definition—Protected Assets

An important issue is to identify what should be protected. At the beginning of the security problem definition process, different kinds of passive entities, *i.e.*, assets requiring protection, should be identified. Please note that assets may be placed within the TOE or in its environment. To elaborate the assets specifications, the predefined generics from Table 8 can be selected. To express the given issue more adequately, the refinement operation can be applied.

The basic asset is data sampled by the intelligent sensor, which can be expressed by the previously defined enhanced generic, here refined:

**Table:** 

*DTO.SensorData.*	*Data measured, stored, processed, or transmitted by an intelligent sensor and data related to the network services.*
*Refinement:**Data related to the methane concentration sampled in time intervals. The thresholds values related to the alarm generation and the energy switch-off, settable with the use of the calibration keyboard, are also included.*	

The mine environment requires that the gas monitoring should be permanent (in defined time intervals), so continuity aspects are also very important. The MEDIS operations, *i.e.*, measurement, processing and transferring results to the MMS central unit, and the generation of local warnings (voice, light) can be considered as certain services whose continuity and quality can be considered as an asset:

**Table:** 

*DTO.SensorService.*	*Services provided by a sensor: measuring, sampling, processing, transferring data, and displaying its status.*
*Refinement:**Sending/receiving the communication protocol messages, the security related data and generating acoustic alarm signals are included.*	

Please note that the sensor data and operations are important to ensure the safety of the mine personnel and the mine equipment.

An important issue is the sensor identity protection to avoid the sensor replacement by a falsified device. For this reason, the MEDIS sensors are equipped with unique identifiers, which are registered and securely inserted during the manufacturing phase. These identifiers should be protected, so they are considered as an auxiliary asset (assets used to protect other assets):

**Table:** 

*DTO.SensorID.*	*Unique identification number of an intelligent sensor.*

The processing resources are not critical because the sensor microcontroller is well balanced to the required speed of operation and this speed is almost constant. Additionally, the transmission resources are considered as not critical because the sensor can work autonomously and redundant sensors are applied. The basic critical resource is energy which can be expressed in the following way:

**Table:** 

*DTO.NodePowerRes.*	*Energy supplying a sensor node.*
*Refinement:**This concerns energy provided by transmission lines from the MMS central station and the energy stored in the battery within the sensor.*	

MEDIS co-operates closely with the supervising entity which stores sampled data and issues commands to the sensor (local mode of operation, though possible, is atypical and less effective). This entity can be considered as an external asset:

**Table:** 

*DEO.CentralUnit.*	*The entity supervising and/or monitoring sensors.*

The MEDIS sensor co-operates with two kinds of specialized equipment (the calibration keyboard and a special energy switch to control the power of the mine machines), absent in previous validations. For this reason, the predefined set of CC-related patterns is extended by a new enhanced generic *DEO.Co-operatEquip* (should be added to the Table 8):

**Table:** 

*DEO.Co-operatEquip.*	*The specialized equipment co-operating with the TOE.*

(Note: the presented validation shows the need to define this item and add it to the set of patterns.)

Thanks to the iteration and refinement operations [3] allowed for enhanced generics, both devices can be expressed by one generic only, but twice iterated and each time differently refined:

**Table:** 

*DEO.Co-operatEquip (1).*	*The specialized equipment co-operating with the TOE.*
*Refinement:**The calibration keyboard used by service personnel to check the sensor and set alarm thresholds.*	
*DEO.Co-operatEquip (2).*	*The specialized equipment co-operating with the TOE.*
*Refinement:**The central power switch of the mining machinery (longwall) and lighting.*	

Because such an IT product as MEDIS may influence the safety of the mine personnel and equipment, the evaluation assurance level (not discussed there) declared for it will be probably EAL3 or above. In this case the security of the development process ought to be considered on a more detailed level according to the DVS (Development security) assurance family of the ALC (Life-cycle support) assurance class [1]/Part 3. For this reason, all design related data should be precisely identified and expressed, e.g., with the use of the previously defined item:

**Table:** 

*DES.DesignData.*	*Sensitive project data and documentation.*
*Refinement:**Logical, physical design data of the TOE hardware, software specifications, code and other related documentation, development aids, test data, user data related documentation, material for software development and manufacturing process.*	

Careful and rigorous design, deployment and management of mine monitoring systems improve mine safety and productivity.

### 4.3. Security Problem Definition—Identification of Active Entities

Active entities embrace different actors, including legal users and intruders. They also represent different roles defined in the TOE life-cycle model (ALC class [1]/Part 3). Such models have different phases (e.g., development, manufacturing, maintenance/operation, end of life), activities of particular phases (e.g., hardware component manufacturing, assembling, repairing, security data insertion) and transitions between phases and activities (e.g., product delivery to service workshop, product component delivery for testing). To elaborate the specification of the MEDIS subjects and other active entities, the predefined enhanced generics from Table 9 can be used.

There are two kinds of direct users of the MMS system and its MEDIS sensors. First, there are the underground mining personnel (miners, supervisors, technical maintenance, mining authority), expressed by the first instance of the *SAU.IntellSensorUser* generic:

**Table:** 

*SAU.IntellSensorUser(1).*	*Authorized entity (user, process) who/which directly or indirectly uses the intelligent sensor.*
*Refinement:**The underground mine personnel, i.e., miners, supervisors, technical maintenance, mining authority.*	

The second group of users embraces mine dispatchers (please note the second instance of the *SAU.IntellSensorUser* generic) and system administrators, who are mainly the surface personnel:

**Table:** 

*SAU.IntellSensorUser(2).*	*Authorized entity (user, process) who/which directly or indirectly uses the intelligent sensor.*
*Refinement:**The surface personnel—mine dispatcher.*	
*SAU.SensorNetAdmin.*	*Authorized administrator of the sensor network and applications.*

The third group of generics expresses the authorized personnel engaged in the development-, manufacturing- and service processes running outside the MEDIS operational environment:

**Table:** 

*SAU.Developer.*	*Personnel involved in the design phase (hardware/software designer, programmer, test engineer).*
*SAU.ManufPers.*	*Personnel involved in the manufacturing processes (components manufacturing, assembly, security data insertion, storage, distribution, repair).*
*SAU.ServicePers.*	*Personnel involved in sensor or sensors system maintenance (storage, installation, inspection, calibration, repair).*
*Refinement:**Personnel can work both in the mine underground and in authorized workshops on the mine surface.*	

Apart from authorized entities, the intruders can be considered. The experience (aftermath catastrophes analyses) shows that the mining personnel in the operational environment should also be considered as intruders. Please note that they are expressed by *SAU.IntellSensorUser(1)*.

Some intruders may operate on the monitoring system level. They can get illegal access and make breaches locally or through other entities co-operating with the MMS systems (e.g., if they are hacked). They can be expressed by the standard enhanced generic:

**Table:** 

*SNA.HighPotIntruder.*	*Attacker having high level skills, enough resources and deep motivation to perform a deliberate attack.*

Numerous and varied non-human, technical or natural causes of harmful events should be taken into account in the mine environment. Generally, they are hard to predict and preventing measures are usually applied against them (represented by security objectives, discussed later). All these causes are expressed by one enhanced generic:

**Table:** 

*SNH.ForceMajeure.*	*Different kinds of unforeseen reasons of harmful events.*
*Refinement:**Outbursts, shocks, water, oil, temperature, vibrations, dust, technical failures,* etc.*—generally all factors or potential causes of problems, specific to the heavy duty mine environment.*	

With respect to the design data, intruders operating within the development-, manufacturing- and maintenance environment can be also identified and expressed (optionally) using the predefined generic:

**Table:** 

*SNA.IndustIntrud.*	*Industry spy or intruder trying to get or counterfeit the design data.*

### 4.4. Security Problem Definition—Identification of Threats

The TOE security problem is mainly expressed by threats. Three kinds of threats can be distinguished:

- threats encompassing direct attacks against the TOE—drawn from Table 10;
- threats related to the operational environment, especially attacks focused on the sensor operations (services) and data sampled, stored, processed and transmitted by the intelligent sensor—drawn from Table 11;
- threats related to the development-, manufacturing- and maintenance processes (specified optionally)—drawn from Table 12.

For each of the identified threats expressing an elementary security problem, the solution is proposed in the form of security objectives countering this threat (selected from Table 15) and specified for the TOE, its operational environment and, optionally, for its site.

Aftermath investigations of methane explosions in mines and mine authority inspections show that the sensor tampering, manipulations and negligence can be considered as causes of catastrophes. A very important issue for right monitoring the mine environment is the sensors placement with respect to the physical properties of methane, conditions in a given location and ventilation system. The first threat concerns a direct attack against data sampled (measured) by sensors, though without breaching the sensor integrity. An enhanced generic, presenting this issue, is additionally refined by adding some details specific to the mine environment.

**Table:** 

*TDA.DisruptSampling.*	*Users or intruders [Sparam <= SAU.IntellSensorUser(1)] could try to manipulate the sensor input [Dparam <= DTO.SensorData] causing wrong input data.*
*Refinement:**The manipulations may concern: the wrong location of the sensor with respect to the predicted methane concentration (*e.g.*, in the air stream enforced by the ventilation system), clogging up the sensor gas entry.*	

A tampering attack can be very dangerous. An intruder may intentionally compromise the physical or logical TOE integrity to counterfeit or destroy the sensor data (any kind of data) and/or disrupt the intelligent sensor operation:

**Table:** 

*TDA.Tamper.*	*Intruder [Sparam <= SAU.IntellSensorUser(1)] physically/logically compromises a sensor node to get assets [Dparam <= DTO.SensorData] or disrupt the node operation [Dparam <= DTO.SensorService].*

Please note that the MEDIS sensor is an element of the mine safety system. Any sensor security problem may cause a mine safety problem, and the safety of the mine personnel and equipment is the key issue. It can be expressed by the threat:

**Table:** 

*TEO.SafetyProblem.*	*The manipulation (corrupting, replaying, blocking) by [Sparam <= SAU.IntellSensorUser(1), SNA.HighPotIntruder] of the data [Dparam<= DTO.SensorData, DTO.SensorService] sampled by sensors with the intention to hide or delay the detection of safety-critical faults in the safety-critical equipment to potentially induce hazards.*

These three main threats are countered by the security objectives concerning the tamper-resistance, data verification, audit, reliable solutions and sensors inspections.

Security objectives declared for the TOE:

**Table:** 

*OINT.TamperResistance.*	*The TOE guarantees its own physical/logical integrity. The means of detecting physical tampering must be provided (*e.g., *seals, tampering detection, special reinforced cases, intrinsically safe solutions).*
*Refinement:**All external sensor connections are adequate to the heavy duty mine environment (outbursts, shocks, water, oil, temperature, vibrations, dust, technical failures).*	
*ODEX.CommQuality.*	*Avoidance of interference, blocked communication spaces, using specialized measures against jamming, collision and flooding.*
*Refinement:**Implement dedicated security measures in the used proprietary communication protocol.*	
*OINT.DataVerification.*	*Verify that the data [Dparam <= DTO.SensorData] are valid.*
*Refinement:**Data sampled, stored, processed and transmitted.*	
*OAVB.Reliability.*	*The sensor must provide reliable service. Applying fault tolerance methods and techniques.*
*OINT.Processing.*	*The sensor must ensure that the processing of input to derive output data [Dparam] is accurate.*
*OADT.Audit.*	*The sensor must audit attempts to undermine its security and should trace them to the associated entities.*

Security objective declared for the TOE operational environment:

**Table:** 

*OSMN.RegularInpections.*	*The sensor must be periodically inspected and calibrated (if necessary).*

The threat related to the node replacement by a counterfeited one should also considered.

**Table:** 

*TDA.ReplaceNode.*	*Attacker [Sparam <= SNA.HighPotIntruder] steals (disables) operational nodes and replaces them with the malicious ones of falsified identities.*

Security objectives declared for the TOE:

**Table:** 

*OIDA.ControlID.*	*Using the properly managed unique identifiers of sensors [Dparam <= DTO.SensorID].*
*OAVB.DataFreshness.*	*Ensure that the message received [Dparam <= DTO.SensorService] is the message sent by the authorized source [Sparam <= DEO.CentralUnit] but not a replayed message sent by the intruder [Sparam <= SNA.HighPotIntruder].*
*OADT.Audit.*	*The sensor must audit attempts to undermine its security and should trace them to the associated entities.*

Security objective declared for the TOE operational environment:

**Table:** 

*OSMN.NetAdmin.*	*Network administration and security policy procedures implementation.*

In the mine environment different unforeseen troubles may occur, caused by non-human factors:

**Table:** 

*TDA.Faults.*	*Faults caused by [Sparam <= SNH.ForceMajeure] in hardware, software, communication procedures could place the sensor node in unforeseen conditions compromising its security.*

Security objectives declared for the TOE:

**Table:** 

*OINT.TamperResistance.*	*The TOE guarantees its own physical/logical integrity. The means of detecting physical tampering must be provided (*e.g., *seals, tampering detection, special reinforced cases, intrinsically safe solutions).*
*Refinement:**All external sensor connections are adequate to the heavy duty mine environment (outbursts, shocks, water, oil, temperature, vibrations, dust, technical failures).*	
*OAVB.Reliability.*	*The sensor must provide reliable service. Applying fault tolerance methods and techniques.*
*OADT.Audit.*	*The sensor must audit attempts to undermine its security and should trace them to the associated entities.*

Security objectives declared for the TOE operational environment:

**Table:** 

*OAVB.RedundNodes.*	*Apply redundant sensor nodes, allowing to lose nodes without any impact on the network (or network application) behavior as a whole.*

The threats concerning manipulation of power and limited power resources can be considered together:

**Table:** 

*TDA.PowerSupply.*	*Users or intruders [Sparam <= SAU.IntellSensorUser(1)] defeat the sensor security by modifying (cutting, reducing, increasing) its power supply [Dparam <= DTO.NodePowerRes].*
*TDA.LimitResour.*	*Exploiting vulnerability related to the node limited resources [Dparam <= DTO.NodePowerRes], an intruder [Sparam <= SAU.IntellSensorUser(1), SNA.HighPotIntruder] causes misbehavior, disconnection or faults within the system.*

Security objectives declared for the TOE:

**Table:** 

*OAVB.ResUnderControl.*	*Control resource [Dparam <= DTO.NodePowerRes].*
*OINT.TamperResistance.*	*The TOE guarantees its own physical/logical integrity. The means of detecting physical tampering must be provided (*e.g., *seals, tampering detection, special reinforced cases, intrinsically safe solutions).*
*Refinement:**All external sensor connections are adequate to the heavy duty mine environment (outbursts, shocks, water, oil, temperature, vibrations, dust, technical failures).*	
*OAVB.Reliability.*	*The sensor must provide reliable service. Applying fault tolerance methods and techniques.*
*OADT.Audit.*	*The sensor must audit attempts to undermine its security and should trace them to the associated entities.*

Security objectives declared for the TOE operational environment:

**Table:** 

*OAVB.RedundNodes.*	*Apply redundant sensor nodes, allowing to lose nodes without any impact on the network (or network application) behavior as a whole.*
*OSMN.RegularInpections.*	*The sensor must be periodically inspected and calibrated (if necessary).*

The entire MMS system can be considered as a specialized hierarchical network with the MMS central unit at the top, cooperating with other IT systems (SCADA, ERP, Management/ Decision Support, *etc.*), and MEDIS and other kinds of sensors at the bottom. For such a complex system it is possible to perform an illegal access type attack against the MEDIS sensor from the MMS central unit side, e.g., “owned” by a hacker or by an untrustworthy person. Illegal access can be considered with respect to the sensor calibration as well. The authorized service personnel are able not only to set wrong alarm thresholds (see *TEO.SecDataAdmin* considered below) but also to manipulate the sensor internal data or functions with the use of the calibration keyboard. Both aspects of illegal access are expressed as follows:

**Table:** 

*TDA.Access.*	*Users or intruders [Sparam <= SNA.HighPotIntruder, SAU.ServicePers] could try to access functions or data [Dparam <= DTO.SensorService, DTO.SensorData] they are not allowed to.*

Security objectives declared for the TOE:

**Table:** 

*OACC.Access.*	*The sensor must control access of connected entities.*
*OIDA.ControlID.*	*Using the properly managed unique identifiers of sensors [Dparam <= DTO.SensorID].*
*Refinement:**Calibration keyboards are also provided with the unique identifiers which can be checked.*	
*OADT.Audit.*	*The sensor must audit attempts to undermine its security and should trace them to the associated entities.*

Security objectives declared for the TOE operational environment:

**Table:** 

*OSMN.NetAdmin.*	*Network administration and security policy procedures implementation.*

For the here discussed complex sensor system, the insufficiencies in the system management, including sensors calibration and controlling the sensor identifiers, may cause serious problems. This issue can be expressed in the following way:

**Table:** 

*TEO.SecDataAdmin.*	*Insufficient administration of the network and security related data [Dparam] of the sensor network by [Sparam <= SAU.SensorNetAdmin, SAU.ServicePers].*
*Refinement:**Includes also the improper calibration and setting up the alarm thresholds.*	

Security objectives declared for the TOE operational environment:

**Table:** 

*OSMN.NetAdmin.*	*Network administration and security policy procedures implementation.*

The last threats deal with development, manufacturing and service. The design-related data can be eavesdropped and abused in different ways, e.g., to prepare attacks in the TOE operational environment or to get know-how by dishonest competitors:

**Table:** 

*TES.Design.*	*Users or intruders [Sparam <= SAU.Developer, SAU.ManufPers, SAU.ServicePers, SNA.IndustIntrud] could try to gain illicit knowledge of the design [Dparam <= DES.DesignData] either from the manufacturer's materials (through theft, bribery, illegal access to IT resources) or from reverse engineering.*

The second issue concerns the troubles caused by improper testing:

**Table:** 

*TES.Test.*	*The use of non-invalidated test modes by [Sparam <= SAU.Developer, SAU.ManufPers, SAU.ServicePers] or not detected backdoors could compromise the sensor security.*

All these problems can be solved by the implementation of technical, physical and organizational measures within the site where the TOE is developed, manufactured and maintained.

Security objective declared optionally for the TOE site processes:

**Table:** 

*OSMN.SiteProcess.*	*Site processes encompassing the development-, manufacturing- and maintenance activities in the life cycle are properly defined, implemented and managed.*

### 4.5. Security Problem Definition—Organizational Security Policies (OSPs)

The security problem definition can be expressed by specifying organizational security policies (OSPs), e.g., using predefined items from Table 13 (supplemented by new defined items). In practice, the main security problem is expressed by threats and OSPs, having auxiliary, supporting meaning. OSPs are used to express issues which are very specific and hard to express by threats. Generally, such an approach allows to get more precise and compact security targets. For each of the identified OSPs expressing an elementary security problem, a solution is proposed in the form of security objectives enforcing the OSP (selected from Table 15) and specified for the TOE, its operational environment, and optionally, for its site processes.

With respect to the methane and coal-dust explosion hazards in the MEDIS operation environment, three OSP rules concerning legal or technical compatibility are specified.

**Table:** 

*PINT.IntrinsicSafe.*	*The intelligent sensor should be intrinsically safe.*

Security objective enforcing the OSP within the TOE:

**Table:** 

*OINT.IntrinsicSafe.*	*The intelligent sensor should be intrinsically safe.*

(Note: the presented validation shows the need to define this item and add it to the set of patterns.)

**Table:** 

*PSMN.ATEX.*	*Ensure compliance with the ATEX equipment directive 94/9/EC.*

(Note: the presented validation shows the need to define this item and add it to the set of patterns.); the ATEX concerns equipment and protective systems used in potentially explosive atmospheres [22].

Security objective enforcing the OSP within the TOE:

**Table:** 

*OSMN.ATEX.*	*The IT product should comply with the ATEX equipment directive 94/9/EC.*

(Note: the presented validation shows the need to define this item and add it to the set of patterns.)

**Table:** 

*PSMN.LegalAct.*	*Ensure compliance with mining legal regulations.*

(Note: the presented validation shows the need to define this item and add it to the set of patterns.)

Security objective enforcing the OSP within the TOE:

**Table:** 

*OSMN.LegalAct.*	*The IT product should comply with the national regulations issued by mining authorities.*

(Note: the presented validation shows the need to define this item and add it to the set of patterns.)

These security objectives are closely related to the intelligent sensor implementation, so they are declared for the TOE.

For the MEDIS security target one main security policy rule, describing the right operation (availability) of the sensor system, is declared:

**Table:** 

*PAVB.SensorSysMain.*	*The data [Dparam <= DTO.SensorData] transmitted by the sensor must be available to the supervising- or data sampling unit [Dparam <= DEO.CentralUnit] so as to allow the unit to determine fully and accurately the sampled data.*

Security objective enforcing the OSP within the TOE operational environment:

**Table:** 

*OAVB.SensorSysMain.*	*The data [Dparam <= DTO.SensorData] sampled by the sensor-based acquisition/monitoring system [Dparam <= DEO.CentralUnit] checked by control authorities must be available and reflect fully and accurately the system objectives.*

It concerns the entire system, so this objective is declared for the TOE operational environment.

To protect the design data and site processes, a general policy is declared (optional):

**Table:** 

*PSMN.ISO27001.*	*Within the sensor development and manufacturing environment the ISMS (Information Security Management System) should be implemented according to ISO/IEC 27001.*

Security objective enforcing the OSP within the TOE site processes:

**Table:** 

*OSMN.ISO27001.*	*Within the sensor development and manufacturing environment the ISMS (Information Security Management System) should be implemented according to ISO/IEC 27001.*

(Note: the presented validation shows the need to define this item and add it to the set of patterns.)

### 4.6. Security Problem Definition—Assumptions

The security problem definition is supplemented by a set of assumptions. The predefined enhanced generics for declaring assumptions are placed in Table 14. For each of the identified assumptions expressing an elementary security issue, a solution is proposed in the form of security objectives upholding the assumption (selected from Table 15) and specified for the TOE operational environment, and optionally, for its site processes.

The first group of assumptions concerns the intended use of the TOE and right management in the operational environment:

**Table:** 

*APR.IntendedUse.*	*The TOE will be used to perform a task or function for which it was designed by [Sparam <= SAU.IntellSensorUser(1)].*

This security objective upholds assumption within the TOE operational environment:

**Table:** 

*OSMN.UserAwarn.*	*User awareness, proper operation regulations and procedures.*

**Table:** 

*APR.TrustAdmin.*	*One or more authorized administrators [Sparm <= SAU.SensorNetAdmin] are assigned who are competent to manage the TOE and the security of the information it contains, and who can be trusted not to deliberately abuse their privileges so as to undermine security.*

This security objective upholds assumption within the TOE operational environment:

**Table:** 

*OSMN.SecManAdmin.*	*The TOE will provide facilities to enable an authorized administrator [Sparam <= SAU.SensorNetAdmin] to effectively manage the TOE and its security functions, and will ensure that only authorized administrators are able to access such functionality.*

The second group of assumptions deals with the development-, manufacturing- and maintenance processes performed in the site (declared optionally):

**Table:** 

*APR.Development.*	*Sensor developers [Sparam <= SAU.Developer] must ensure that the assignment of responsibilities during the development is done in a manner which maintains IT security.*
*APR.Manufacturing.*	*Sensor manufacturers [Sparam <= SAU.ManufPers] must ensure that the assignment of responsibilities during manufacturing is done in a manner which maintains IT security, and that during the manufacturing process the sensor is protected against physical attacks which might compromise IT security. All testing facilities for the manufacturing phase (test points, commands) should be removed or disabled before delivery.*
*APR.Delivery.*	*Sensor manufacturers, fitters, workshops [Sparam <= SAU.ManufPers, SAU.ServicePers] must ensure that handling of the sensor is done in a manner which maintains IT security.*
*APR.ApprovedWorkshops.*	*Installation, calibration and repair of the sensor and its monitoring unit must be carried out by trusted and approved fitters or workshops by the authorized [Sparam <= SAU.ServicePers].*

This security objective upholds assumptions within the TOE site processes:

**Table:** 

*OSMN.SiteProcess.*	*Site processes encompassing the development-, manufacturing- and maintenance activities in the life cycle are properly defined, implemented and managed.*

### 4.7. Resume Concerning Declared Security Objectives

As was mentioned earlier (subsection 4.4), the particular security objectives express elementary solutions of the elementary security problems.

Some security objectives are declared for the TOE and then the TOE is responsible for a given elementary solution, *i.e.*, countering the threat and/or enforcing the OSP. These TOE security objectives are transformed later to security functional requirements (SFRs) expressed with the use of functional components from Part 2 of [1], and later implemented in the security functions within the TOE on the claimed EAL, and evaluated. Thirteen TOE security objectives are identified and around them security functions will be developed. These are the following:

- ten items countering threats:

**Table:** 

*OINT.TamperResistance*,	*ODEX.CommQuality*,	*OINT.DataVerification*,
*OAVB.Reliability*,	*OINT.Processing*,	*OADT.Audit*,
*OIDA.ControlID*,	*OAVB.DataFreshness*,	*OAVB.ResUnderControl*,
*OACC.Access*,
- three items enforcing OSPs:

**Table:** 

*OINT.IntrinsicSafe*,	*OSMN.ATEX*,	*OSMN.LegalAct*.

Some security objectives are declared for the TOE operational environment, which means that this environment is responsible for the elementary security solution, *i.e.*, for countering the given threat, and/or enforcing the OSP, as well as holding the assumption. The security objectives declared for the TOE operational environment are not transformed to the SFRs. They are envisaged in the TOE documentation used as evidences elaborated for certification, and in the security mechanisms implemented within the TOE operational environment. For MEDIS, six objectives for the TOE operational environment are identified:

**Table:** 

*OSMN.RegularInpections*,	*OSMN.NetAdmin*,	*OAVB.RedundNodes*,
*OAVB.SensorSysMain*,	*OSMN.UserAwarn*,	*OSMN.SecManAdmin*.

Moreover, two security objectives are declared optionally for the site processes:

**Table:** 

*OSMN.SiteProcess*,	*OSMN.ISO27001*.

They are mainly related to the evidences elaborated for the ALC_DVS and AVA_VAN assurance families [1]/Part 3.

During the evaluation related to the Common Criteria certification, the TOE operational environment objectives and the TOE site objectives are considered as dogmas.

## 5. Conclusions

### 5.1. Validation Summary

The validation of the CC-related design patterns on the MEDIS sensor example shows that most of the identified security issues can be expressed with the use of predefined patterns, though several new items were defined to express specific issues related to the considered domain of application. The following seven patterns are defined as a result of the performed validation:

- to add to Table 8 as an asset related to the sensor operational environment:
  “*DEO.Co-operatEquip. The specialized equipment co-operating with the TOE.*”
- to add to Table 13 as OSPs related to the TOE:
  “*PSMN.ATEX. Ensure compliance with the ATEX equipment directive 94/9/EC.*”
  “*PSMN.LegalAct. Ensure compliance with mining legal regulations.*”
- to add to Table 15 as security objectives concerning mainly the TOE:
  “*OINT.IntrinsicSafe. The intelligent sensor should be intrinsically safe.*”
  “*OSMN.ATEX. The IT product should comply with the ATEX equipment directive 94/9/EC.*”
  “*OSMN.LegalAct. The IT product should comply with the national regulations issued by mining authorities.*”
- to add to Table 15 as security objectives concerning mainly the site processes:
  “*OSMN.ISO27001. Within the sensor development and manufacturing environment the ISMS (Information Security Management System) should be implemented according to ISO/IEC 27001.*”

During validation all security issues are expressed without changing semantics of predefined enhanced generics. To express very specific issues, the refinement operation on a pattern was enough.

### 5.2. Method Evaluation and Future Development

The intelligent sensors and network-based software systems which integrate them:

- are considered as “IT products and systems” with all security issues inherent to them,
- are used often for high risk applications which require right assurance.

These factors allow discussing the intelligent sensors and sensor systems as a Common Criteria domain of application. This domain has emerging character. The exhaustive review of the state-of-the-art of this domain [3] presents this situation in details.

The paper presents the evaluation of the method supporting the sensor developers in the use of the Common Criteria methodology. The method, based on the motion sensors for digital tachographs project [5] was introduced [3] and evaluated on the intelligent sensor for medical applications. This paper presents a slightly improved version of the method and its validation on another, very specific sensor designed for mining applications.

The general contribution of the paper, related to [3], deals with:

- defining CC-related security design patterns, called here enhanced generics, to express elementary security problems (*i.e.*, threats, security organizational policies, assumptions), elementary solutions of these problems (*i.e.*, security objectives), and implementation of these solutions (*i.e.*, security functions based on requirements) with respect to the intelligent sensors needs and the life-cycle model,
- providing knowledge how to apply these patterns to elaborate Common Criteria complaint security models, called security targets, for a broader class of intelligent sensors.

The direct contribution of the paper is concisely presenting, evaluating and improving the proposed patterns-based method and showing how to apply it to one of the emerging domains of application related to mining (early detection of methane hazards).

Generally, this method has open character—new security patterns can be always added, still the author’s experience shows that uncontrolled extension of the existing set of patterns decreases coherency and quality of the elaborated specifications. Instead of defining numerous items, the enhanced generics features are used, like parameterization, refinement and iteration. The quantity of patterns should be necessary and sufficient to describe security features of a given domain of application. To achieve this aim, the method validation on several design examples will be suitable. Each validation means new experience allowing to complete a given set of patterns for an additional kind of sensors as well as to improve quality and preciseness of security specifications provided by the method. These improvements allow applying the knowledge engineering methodology and tools. The next, planned step is to develop an ontology of the CC-related security design patterns and knowledge base supporting the intelligent sensors developers. The initial version of this knowledge base restricted to the motion sensors is presented in [16].

The presented intelligent sensors development patterns-based method is focused on the use of the matured but still improved Common Criteria methodology providing assurance for IT products or systems, including sensor systems. The formalized, rigorous development and independent security evaluation are the basis of this assurance. IT products or systems can get worldwide recognized certificates and can be used in higher risk applications.

Elaborating a Common Criteria compliant method dedicated for the intelligent sensors should become widespread in the secure intelligent sensors applications. The review of this application domain was provided in [3]. The most relevant currently running researches concern:

- airplane health monitoring systems,
- safety-critical assets distribution systems,
- motion sensors of digital tachographs,
- SCADA (Supervisory Control And Data Acquisition) related products,
- specialized firewalls used in control and automation systems, co-operating with sensor networks, but the most numerous group of the certified solutions [5,16] concerns digital tachograph applications, e.g., [13]. In the near future new certification processes in this domain are expected because the document [14] has been recently revised. The Common Criteria methodology use allows to avoid shortage related to risk factors in the operational and development environments. First and foremost, threats and vulnerabilities are exhaustively identified and analyzed if they are properly countered. Independent evaluation supports these activities.

## Figures and Tables

**Table 1.** Common Criteria related terms and acronyms [1]/Part1.

Acronym	Meaning
assets	Entities that the owner of the TOE presumably places value upon.
assurance	Grounds for confidence that a TOE meets the SFRs.
attack potential	A measure of the effort to be expended in attacking a TOE, expressed in terms of an attacker's expertise, resources and motivation.
class	A grouping of CC families that share a common focus.
component	The smallest selectable set of elements on which requirements may be based.
connectivity	The property of the TOE which allows interaction with IT entities external to the TOE. This includes exchange of data by wire or by wireless means, over any distance in any environment or configuration.
development environment	The environment in which the TOE is developed.
element	An indivisible statement of security need.
evaluation	Assessment of a PP, an ST or a TOE, against defined criteria.
evaluation assurance level (EAL)	An assurance package, consisting of assurance requirements drawn from CC Part 3, representing a point on the CC predefined assurance scale.
family	A grouping of components that share a similar goal but may differ in emphasis or rigor.
formal	Expressed in a restricted syntax language with defined semantics based on well-established mathematical concepts.
identity	A representation (e.g., a string) uniquely identifying an authorized user, which can either be the full or abbreviated name of that user or a pseudonym.
informal	Expressed in natural language.
iteration	The use of the same component to express two or more distinct requirements.
life-cycle	The sequence of stages of existence of an object (for example a product or a system) in time.
life-cycle model	Description of the stages and their relations to each other that are used in the management of the life-cycle of a certain object, how the sequence of stages looks like and which high level characteristics the stages have.
object	A passive entity in the TOE, that contains or receives information, and upon which subjects perform operations.
operation (on a component of the CC)	Modifying or repeating that component. Allowed operations on components are assignment, iteration, refinement and selection.
operation (on an object)	A specific type of action performed by a subject on an object.
operational environment	The environment in which the TOE is operated.
organizational security policy (OSP)	A set of security rules, procedures, or guidelines imposed (or presumed to be imposed) now and/or in the future by an actual or hypothetical organization in the operational environment.
package	A named set of either functional or assurance requirements (e.g., EAL 3).
protection profile (PP)	An implementation-independent statement of security needs for a TOE type.
refinement	The addition of details to a component.
SAR	Security assurance requirement.
SFR	Security functional requirement.
security objective	A statement of intent to counter identified threats and/or satisfy identified organization security policies and/or assumptions.
security target (ST)	An implementation-dependent statement of security needs for a specific identified TOE.
semiformal	Expressed in a restricted syntax language with defined semantics.
subject	An active entity in the TOE that performs operations on objects.
target of evaluation (TOE)	A set of software, firmware and/or hardware possibly accompanied by guidance.
TOE security functionality (TSF)	A set consisting of all hardware, software, and firmware of the TOE that must be relied upon for the correct enforcement of the SFRs.
trusted channel	A means by which a TSF and a remote trusted IT product can communicate with necessary confidence.
trusted IT product	An IT product other than the TOE which has its security functional requirements administratively coordinated with the TOE and which is assumed to enforce its security functional requirements correctly (e.g., by being separately evaluated).
trusted path	A means by which a user and a TSF can communicate with necessary confidence.

**Table 2.** Family field defining assets and other passive entities.

Prefix	Meaning
“DTO”	“TOE related assets”
“DEO” (former “DIT”)	“assets within the TOE operational environment”
“DES” (former “DAP”)	“assets within the TOE site”

**Table 3.** Family field defining subjects and other active entities.

Prefix	Meaning
“SAU”	“authorized subject, e.g., user, administrator, process”
“SNA”	“unauthorized entity, e.g., intruder”
“SNH”	“non-human malicious entity, e.g., force majeure, failure”

**Table 4.** Family field defining threats.

Prefix	Meaning
“TDA”	“direct attacks against the TOE”
“TEO” (former “TIT”)	“attacks against the TOE operational environment”
“TES” (former “TPH”)	“attacks against the TOE site”

**Table 5.** Family field concerning assumptions.

Prefix	Meaning
“ACN”	“connectivity aspects”
“APR”	“personnel/organizational aspects”
“APH”	“physical aspects”

**Table 6.** Family field for Organizational Security Policies and security objectives.

Security objective prefix	OSP prefix	Meaning
“OACC”	“PACC”	“access control and information flow control”
“OIDA”	“PIDA”	“identification and authentication”
“OADT”	“PADT”	“accountability and security audit”
“OINT”	“PINT”	“integrity”
“OAVB”	“PAVB”	“availability”
“OPRV”	“PPRV”	“privacy”
“ODEX”	“PDEX”	“data exchange”
“OCON”	“PCON”	“confidentiality”
“OEIT”	“PEIT”	“IT aspects of the TOE operational environment or site”
“OEPH”	“PEPH”	“technical/physical aspects of the TOE operational environment or site”
“OSMN”	“PSMN”	“security maintenance”

**Table 7.** Family field related to the security functions.

Prefix	Meaning
“SFAU”	“security functions dealing with audit”
“SFCO”	“security functions dealing with communication”
“SFCS”	“security functions dealing with cryptographic support”
“SFDP”	“security functions dealing with user data protection”
“SFIA”	“security functions dealing with identification and authentication”
“SFMT”	“security functions dealing with security management”
“SFPR”	“security functions dealing with privacy issues”
“SFPT”	“security functions dealing with the protection of the TSF”
“SFRU”	“security functions dealing with resource utilization”
“SFTA”	“security functions dealing with the TOE access”
“SFTP”	“security functions dealing with trusted path/channels”

**Table 8.** Enhanced generics representing protected assets and other passive entities.

Prefix, mnemonic	Description
Basic assets	
*DTO.SensorData*	*Data measured, stored, processed, or transmitted by an intelligent sensor and data related to the network services.*
*DTO.SensorService*	*Services provided by a sensor: measuring, sampling, processing, transferring data, and displaying its status.*
Assets related to the sensor availability	
*DTO.NodePowerRes*	*Energy supplying a sensor node.*
*DTO.NodeProcesRes*	*Node processing ability.*
*DTO.NodeTransmRes*	*Node data transmission ability.*
Security-related data	
*DTO.SensorID*	*Unique identification number of an intelligent sensor.*
*DTO.Password*	*Password for authentication.*
*DTO.CryptoKey*	*Symmetric cryptographic key*
*DTO.Credent*	*Credential or shared secret.*
*DTO.RndNumber*	*Random number to derive a cryptographic key.*
*DTO.PubKey*	*Public key.*
*DTO.PrivKey*	*Private key.*
*DTO.BioData*	*Biometric or physiological data used for the identification of a person.*
Assets related to the sensor operational environment	
*DEO.BaseStation*	*A distinguished node of a wireless sensor network (WSN) used to control the network or as a gateway intermediating between WSN and other network.*
*DEO.CentralUnit*	*The entity supervising and/or monitoring sensors.*
*DEO.NeighborSenNode*	*A neighbor node whose security mutually relies on the considered sensor node.*
*DEO.SampledDataBase*	*Data sampled by sensors and stored in a common data base on the distinguished server.*
Assets related to the sensor site processes (considered optionally)	
*DES.DesignData*	*Sensitive project data and documentation.*

**Table 9.** Enhanced generics representing subjects, intruders and other active entities.

Prefix, mnemonic	Description
Legal users	
*SAU.IntellSensorUser*	*Authorized entity (user, process) who/which directly or indirectly uses the intelligent sensor.*
*SAU.SensorNetAdmin*	*Authorized administrator of the sensor network and applications.*
Legal actors participating in the life cycle processes (considered optionally)	
*SAU.Developer*	*Personnel involved in the design phase (hardware/software designer, programmer, test engineer).*
*SAU.ManufPers*	*Personnel involved in the manufacturing processes (components manufacturing, assembly, security data insertion, storage, distribution, repair).*
*SAU.ServicePers*	*Personnel involved in sensor or sensors system maintenance (storage, installation, inspection, calibration, repair).*
Intruders	
*SNA.HighPotIntruder*	*Attacker having high level skills, enough resources and deep motivation to perform a deliberate attack.*
*SNH.ForceMajeure*	*Different kinds of unforeseen reasons of harmful events.*
Intruders relevant to the site processes (considered optionally)	
*SNA.IndustIntrud*	*Industry spy or intruder trying to get or counterfeit the design data.*

**Table 10.** Direct attacks against the intelligent sensor (TOE)—threats expressed by enhanced generics.

Prefix, mnemonic	Description
Threats against data sampled/measured by the sensor (TOE)	
*TDA.DisruptSampling*	*Users or intruders [Sparam] could try to manipulate the sensor input [Dparam] causing wrong input data.*
Direct attacks against data stored, processed and transferred by the sensor (TOE)	
*TDA.NodeCompromise*	*Attacker [Sparam] can: eavesdrop the traffic, inject packets or replay older messages, because wireless communication generally is not secure. After the node compromising, all information it holds [Dparam] is known to the attacker and/or node operation or communication is broken [Dparam].*
*TDA.Access*	*Users or intruders [Sparam] could try to access functions or data [Dparam] they are not allowed to.*
*TDA.SensorDataEavsdrop*	*Intruder [Sparam] eavesdrops sensor data [Dparam].*
*TDA.SensDataMdfy*	*Intruder [Sparam] modifies sensor data (falsifies them) [Dparam].*
Threats exploiting vulnerabilities related to the restricted resources	
*TDA.LimitResour*	*Exploiting vulnerability related to the node limited resources [Dparam], an intruder [Sparam] causes misbehavior, disconnection or faults within the system.*
Threats against the sensor identity	
*TDA.CloneNode*	*Attacker [Sparam] duplicates an operational node causing that both nodes (i.e.*, *original and duplicated one of the same identity) are able to communicate with the given node.*
*TDA.ReplaceNode*	*Attacker [Sparam] steals (disables) operational nodes and replaces them with the malicious ones of falsified identities.*
*TDA.FabricateNode*	*Attacker [Sparam] adds a malicious node with fabricated identity to the network as an operational node.*
*TDA.SybilAttack*	*Attacker [Sparam] adds a malicious node presenting multiple identities, as if it were multiple nodes to control a considerable part of the system, breaching its redundancy.*
Attacks against the security-related data	
*TDA.PowAnalys*	*The power consumption of some microprocessors causes leakage of information during certain cryptographic operations. The attacker [Sparam] uses this information to substantially reduce the key space that needs to be considered in a brute-force search for the secret key [Dparam].*
*TDA.SecDataLeakage*	*The attacker [Sparam] causes that security sensitive data (authentication data, keys, credentials* etc.*) [Dparam] leak out from the TOE.*
Threats dealing with the sensor integrity	
*TDA.Tamper*	*Intruder [Sparam] physically/logically compromises a sensor node to get assets [Dparam] or disrupt the node operation [Dparam].*
*TDA.EnvironAttack*	*Intruders [Sparam] could compromise the sensor security through environmental attacks (thermal, electromagnetic, optical, chemical, mechanical).*
*TDA.PowerSupply*	*Users or intruders [Sparam] defeat the sensor security by modifying (cutting, reducing, increasing) its power supply [Dparam].*
Unforeseen natural catastrophes, emergencies and failures	
*TDA.Faults*	*Faults caused by [Sparam] in hardware, software, communication procedures could place the sensor node in unforeseen conditions compromising its security.*

**Table 11.** Threats related to the TOE operational environment expressed by enhanced generics.

Prefix, mnemonic	Description
Attacks against sensor network and transmission ability (TOE operational environment)	
*TEO.CommInterfer*	*Intruder [Sparam] interferes network communication by sending messages through different protocol layers (*e.g., *jamming, collisions, flooding), causing that the data [Dparam] are lost (usually inconspicuously for their owner or destination) or the node disappears (“has been stolen”).*
*TEO.RoutingMisuse*	*Intruder [Sparam] interferes routing by ignoring all or some messages or network communication.*
*TEO.Malware*	*Malicious software designed to infiltrate or damage a sensor node or sensor network.*
*TEO.BackdoorOpen*	*Using the wireless access point an intruder [Sparam] creates a backdoor for the network of an organization (corporation) from the sensor node side.*
*TEO.AttackPropagation*	*The possibility of attack propagation from a node to other nodes, gateways and external servers.*
*TEO.UncontrolledArea*	*Due to uncontrolled pervasiveness of a wireless sensors network, the node works in an uncontrolled network area accessible to potential intruders [Sparam].*
Attacks causing safety problems	
*TEO.SafetyProblem*	*The manipulation (corrupting, replaying, blocking) by [Sparam] of the data [Dparam] sampled by sensors with the intention to hide or delay the detection of safety-critical faults in the safety-critical equipment to potentially induce hazards.*
Attacks exploiting vulnerabilities related to insufficient administration	
*TEO.SecDataAdmin*	*Insufficient administration of the network and security related data [Dparam] of the sensor network by [Sparam].*

**Table 12.** Threats related to the TOE development-, manufacturing- and maintaining processes expressed by enhanced generics (considered optionally).

Prefix, mnemonic	Description
Attack on the development-, manufacturing- and maintenance environment	
*TES.Design*	*Users or intruders [Sparam] could try to gain illicit knowledge of the design [Dparam] either from the manufacturer's materials (through theft, bribery, illegal access to IT resources) or from reverse engineering.*
*TES.Test*	*The use of non-invalidated test modes by [Sparam] or not detected backdoors could compromise the sensor security.*
*TES.SecurityData*	*Users or intruders [Sparam] could try to gain illicit knowledge of security data [Dparam] during security data generation, transport or storage in the equipment.*
*TES.Software*	*Users or intruders [Sparam] could try to modify the sensor software.*

**Table 13.** Enhanced generics representing Organizational Security Policies.

Prefix, mnemonic	Description
OSPs related to the TOE	
*PSMN.ISA99*	*Ensure compliance with the ISA 99 Manufacturing and Control Systems Security Standard.*
*PINT.IntrinsicSafe*	*The intelligent sensor should be intrinsically safe.*
*PSMN.HIPAA*	*Ensure compliance with the Health Insurance Portability and Accountability Act (HIPAA).*
OSPs related to the TOE operational environment	
*PAVB.SensorSysMain*	*The data [Dparam] transmitted by the sensor must be available to the supervising- or data sampling unit [Dparam] so as to allow the unit to determine fully and accurately the sampled data.*
OSPs related to the site processes (considered optionally)	
*PSMN.ThreatVulnNotif*	*Notification of threats and vulnerabilities according to ISO/IEC 27001/A12.6.1. Appropriate authorities shall be immediately notified of any threats or vulnerabilities impacting systems that process their data.*
*PSMN.ISO27001*	*Within the sensor development and manufacturing environment the ISMS (Information Security Management System) should be implemented according to ISO/IEC 27001.*

**Table 14.** Enhanced generics representing assumptions.

Prefix, mnemonic	Description
Assumptions related to the TOE operational environment	
*APR.IntendedUse*	*The TOE will be used to perform a task or function for which it was designed by [Sparam].*
*APR.TrustAdmin*	*One or more authorized administrators [Sparm] are assigned who are competent to manage the TOE and the security of the information it contains, and who can be trusted not to deliberately abuse their privileges so as to undermine security.*
Assumptions related to the site processes (considered optionally)	
*APR.Development*	*Sensor developers [Sparam] must ensure that the assignment of responsibilities during the development is done in a manner which maintains IT security.*
*APR.Manufacturing*	*Sensor manufacturers [Sparam] must ensure that the assignment of responsibilities during manufacturing is done in a manner which maintains IT security, and that during the manufacturing process the sensor is protected against physical attacks which might compromise IT security. All testing facilities for the manufacturing phase (test points, commands) should be removed or disabled before delivery.*
*APR.Delivery*	*Sensor manufacturers, fitters, workshops [Sparam] must ensure that handling of the sensor is done in a manner which maintains IT security.*
*APR.SecDataGenAlgor*	*Security data generation algorithms must be accessible to authorized and trusted persons only [Sparam].*
*APR.SecDataInsert*	*Security data [Dparam] must be generated, transported, and inserted into the sensor, in such a way to preserve its appropriate confidentiality and integrity by the authorized [Sparam].*
*APR.ApprovedWorkshops*	*Installation, calibration and repair of the sensor and its monitoring unit must be carried out by trusted and approved fitters or workshops by the authorized [Sparam].*
*APR.SoftwareUpgAnal*	*Software revisions must be granted security certification before they can be implemented in the sensor. There is no way to analyze or debug software in the field.*

**Table 15.** Enhanced generics representing security objectives.

Prefix, mnemonic	Description
Security objectives concerning mainly the TOE	
*OAVB.Reliability*	*The sensor must provide reliable service. Applying fault tolerance methods and techniques.*
*OAVB.ResUnderControl*	*Control resource [Dparam].*
*OINT.TamperResistance*	*The TOE guarantees its own physical/logical integrity. The means of detecting physical tampering must be provided (*e.g., *seals, tampering detection, special reinforced cases, intrinsically safe solutions).*
*OINT.Processing*	*The sensor must ensure that the processing of input to derive output data [Dparam] is accurate.*
*OINT.PowAnalResist*	*Solutions resistant to the simple/differential power analysis attacks (SPA/DPA) are implemented.*
*OINT.DataVerification*	*Verify that the data [Dparam] are valid.*
*OINT.AntiMalware*	*Specialized anti-malware software.*
*OIDA.ControlID*	*Using the properly managed unique identifiers of sensors [Dparam].*
*OIDA.Authentication*	*The sensor must authenticate connected entities.*
*OACC.Access*	*The sensor must control access of connected entities.*
*OCON.DataEncrypt*	*Encrypt the data [Dparam].*
*OCON.SecDataProt*	*When a node is turned off, no security material (such as a shared secret or a static public/private key) [Dparam] should have to be stored permanently in the non-volatile memory of the node (a pre-configured shared secret obviously does not satisfy this requirement).*
*OADT.Audit*	*The sensor must audit attempts to undermine its security and should trace them to the associated entities.*
*OSMN.SecManAdmin*	*The TOE will provide facilities to enable an authorized administrator [Sparam] to effectively manage the TOE and its security functions, and will ensure that only authorized administrators are able to access such functionality.*
Security objectives concerning mainly the TOE operational environment	
*OAVB.SensorSysMain*	*The data [Dparam] sampled by the sensor-based acquisition/monitoring system [Dparam] checked by control authorities must be available and reflect fully and accurately the system objectives.*
*OAVB.RedundNodes*	*Apply redundant sensor nodes, allowing to lose nodes without any impact on the network (or network application) behavior as a whole.*
*OAVB.DataFreshness*	*Ensure that the message received [Dparam] is the message sent by the authorized source [Sparam] but not a replayed message sent by the intruder [Sparam].*
*OINT.MajorityVoting*	*Apply the majority voting scheme to determine the validity of an alarm raised by neighboring nodes based on their own measurement.*
*OINT.IdentCapVsNodeCap*	*Testing limited resource (radio communication capability). Assuming that a device can access only one radio channel at a time and checking that each identity has no less capability than a physical node (all identities have channels assigned and must send messages through them simultaneously; the system detects the attack when it receives no message in its channel).*
*OCON.CryptoScheme*	*Applying the cryptographic scheme (key management, operations) with respect to the existing communication resources.*
*OCON.CryptoBoundary*	*Setup the cryptographic boundary inside the TOE from where security sensitive data [Dparam] shall not leak. The boundary encompasses the TOE parts where security sensitive data are generated, stored, updated and used.*
*ODEX.MultipleCommPaths*	*Use redundant communication paths, specialized countermeasures (*e.g., *against blackholes, misdirection, wormholes), and controlling of the routing information.*
*ODEX.CommQuality*	*Avoidance of interference, blocked communication spaces, using specialized measures against jamming, collision and flooding.*
*OEIT.SecPerimVsTrRangeCtrl*	*The physically controlled security perimeter, where nodes are placed, should be defined with respect to the range of wireless transmission.*
*OEIT.IdentPositionVsNodePosition*	*Assuming that no identities are at the same position, checking the identity position* versus *the node position claiming this identity. Sensor measurements are credible when they can be associated with their physical locations.*
*OSMN.RegularInpections*	*The sensor must be periodically inspected and calibrated (if necessary).*
*OSMN.SecDatManag*	*Periodic changes to security data [Dparam] managed by [Sparam].*
*OSMN.NetAdmin*	*Network administration and security policy procedures implementation.*
*OSMN.UserAwarn*	*User awareness, proper operation regulations and procedures.*
*OSMN.PatientSecurity*	*The monitored patient is within the access-restricted area and the medical personnel or household members take care of her/him.*
*OSMN.HIPAA*	*The medical system should comply with the Health Insurance Portability and Accountability Act (HIPAA).*
Security objectives concerning the site processes	
*OSMN.SiteProcess*	*Site processes encompassing the development-, manufacturing- and maintenance activities in the life cycle are properly defined, implemented and managed.*

**Table 16.** TOE security functions—examples related to the motion sensor project [16].

Prefix, mnemonic	Description
*SFAU.AuditFacilities*	*Generating audit records of the events breaching the sensor security*
*SFDP.StoredDataInteg*	*The TSF monitors the integrity of stored data (cryptographic keys, identification data, installation data).*
*SFIA.CryptoIAfacilities*	*The TSF ensures the key-based authentication.*
*SFPT.Selftest*	*The TSF performs self tests (the verification of the security data integrity and the verification of the stored executable code) during the initial start-up, and during normal operation, to verify its correct operation.*
*SFPT.SelfProtection*	*The secure state is preserved when the deviation or cut-off of the power supply as well as the hardware sabotage occur.*
*SFTA.AccCtrl*	*Access control to the functions and data based on the security attributes ensuring that information is read from, created in, or modified into the TOE only by authorized subjects.*
*SFTP.SecDataExch*	*Using the symmetric key cryptography, the TSF protects the confidentiality, authenticity and integrity of data exchanged between the sensor and monitoring unit.*

## References

[1] (2009). Common Criteria for IT Security Evaluation.

[2] Common Criteria Portal Home page.

[3] Bialas A. (2010). Intelligent Sensors Security. Sensors.

[4] Malasri K., Wang L. (2009). Design and Implementation of a Secure Wireless Mote-Based Medical Sensor Network. Sensors.

[5] Bialas A. (2009). Security-Related Design Patterns for Intelligent Sensors Requiring Measurable Assurance. Electr. Rev (Przegląd Elektrotechniczny).

[6] Hermann D.S. (2003). Using the Common Criteria for IT Security Evaluation.

[7] Bialas A. (2008). Semiformal Common Criteria Compliant IT Security Development Framework. Stud. Inf.

[8] Bialas A. Semiformal Framework for ICT Security Development. http://www.8iccc.com/index.php.

[9] Bialas A., Zamojski W., Mazurkiewicz J., Sugier J., Walkowiak T. (2007). Semiformal Approach to the IT Security Development.

[10] Yoshioka N., Washizaki H., Maruyama K. (2008). A survey on security patterns. Prog. Inf.

[11] Jürjens J. (2005). Secure Systems Development with UML.

[12] Schumacher M., Fernandez-Buglioni E., Hybertson D., Buschmann F., Sommerlad P. (2006). Security Patterns: Integrating Security and Systems Engineering.

[13] (2005). Security Target IS2000 Smartach SRES, P206412.

[14] (2002). Commission Regulation (EC) No.1360/2002 on Recording Equipment in Road Transport. Annex 1B Requirements for Construction, Testing, Installation and Inspection. Off. J. Eur. Commun.

[15] (2007). Site Certification. Supporting Document Guidance.

[16] Bialas A. (2009). Ontological Approach to the Motion Sensor Security Development. Electr. Rev (Przegląd Elektrotechniczny).

[17] Figaro (2005). General Information for TGS Sensors.

[18] Wise Control (2005). Gas Detectors and Related Equipment.

[19] EMAG Home page.

[20] Wasilewski S. (2007). Monitoring of Gas Hazards in Polish Underground Hard Coal Mines. Mechanizacja i Automatyzacja Górnictwa.

[21] Wasilewski S. (2009). Integrated Outburst Detection Sensor—Model Tests. Mechanizacja i Automatyzacja Górnictwa.

[22] (2008). EU Directive 94/9/EC—Equipment and Protective Systems Intended for Use in Potentially Explosive Atmospheres (ATEX) Version 2.

